# Evaluation of Loop-Mediated Isothermal Amplification Assay in Diagnosing Tuberculous Meningitis

**DOI:** 10.7759/cureus.57490

**Published:** 2024-04-02

**Authors:** Vishal S Chauhan, Pankaj Jorwal, Binit K Singh, Neeraj Nischal, Manish Soneja, Surabhi Vyas, Venugopalan Y Vishnu, Prayas Sethi, Piyush Ranjan, Naveet Wig

**Affiliations:** 1 Medicine, All India Institute of Medical Sciences, New Delhi, IND; 2 Radiodiagnosis, All India Institute of Medical Sciences, New Delhi, IND; 3 Neurology, All India Institute of Medical Sciences, New Delhi, IND

**Keywords:** mgit 960 culture, cns tb, mtbc, genexpert®, composite reference standard

## Abstract

Background: Resource-limited settings like India need a simple, quick, and temperature-independent point-of-care diagnostic test that can diagnose tuberculous meningitis (TBM) at the earliest.

Methods: A prospective study was carried out at a tertiary care center in North India wherein 50 subjects suspected of TBM were recruited and followed up for six months between January 2019 and December 2020. The aim was to evaluate the performance of loop-mediated isothermal amplification (TB-LAMP) in diagnosing TBM as compared to a composite reference standard (CRS), mycobacteria growth indicator tube 960 (MGIT 960) culture, and GeneXpert^®^.

Results: Out of 50 patients, 32 were TBM cases (64%), and 18 were non-TBM cases (36%). The sensitivity of TB-LAMP and GeneXpert^®^ for TBM diagnosis against CRS was 53% (17/32) for both, and the specificity was 78% (14/18) and 89% (16/18), respectively. On comparing TB-LAMP against GeneXpert^®^ for TBM diagnosis, the two methods had almost perfect agreement (Cohen's kappa=0.83) with statistical significance (p<0.001).

Conclusion: The performance of TB-LAMP assay is comparable to GeneXpert^®^ in diagnosing TBM, and it may be used as a substitute for CSF GeneXpert^®^ in resource-limited settings.

## Introduction

Globally, in 2019, about 10 million people developed tuberculosis (TB), and 1.4 million died because of it. India had the largest share (26%) of these cases [1]. Though the exact prevalence of central nervous system tuberculosis (CNS TB) in India is unknown, it is estimated that it accounts for 1% of all TB cases [2]. Clinically, tuberculous meningitis (TBM) patients usually present with fever, neck stiffness, headache, focal neurological deficit, and altered sensorium [3]. Neck stiffness may be absent in TBM patients during early disease [4]. In addition, nonspecific symptoms like loss of appetite and malaise precede worsening headache and vomiting, thus making early diagnosis difficult, leading to complications and death.

Conventional microbiological methods like acid-fast bacilli (AFB) smear and mycobacteria growth indicator tube (MGIT) 960 culture have low sensitivity or give delayed results [5]. Newer molecular nucleic acid amplification tests (NAATs) have been developed over time for rapid detection like GeneXpert®. The sensitivity of GeneXpert® in TBM diagnosis is around 60%, with specificity approaching 100% [6-8]. WHO has recommended GeneXpert® as a pivotal diagnostic test in suspected patients [9-10]. But most molecular tests, including GeneXpert®, require technical expertise, proper setup, expensive equipment, and temperature maintenance, thus preventing their use in limited-resource settings. LoopAMP™ (LAMP) MTBC detection kit (Eiken Chemical Company Limited, Tokyo, Japan) is a commercially available kit for the detection of *Mycobacterium tuberculosis complex* (MTBC) in sputum specimens. LAMP is an isothermal NAAT that targets six diverse sequences of MTBC DNA. WHO has also recommended LAMP for both sputum AFB-positive and AFB-negative specimens as a replacement test for conventional microscopy to diagnose TB [11]. In low-income countries, which are endemic for TB and have a majority of the population residing in rural areas with constrained resources, there is an unmet need for a test with good diagnostic efficacy and minimal resource requirements. Hence, this prospective study was planned to evaluate the role of loop-mediated isothermal amplification (TB-LAMP) assay in diagnosing TBM.

## Materials and methods

**Figure 1:**
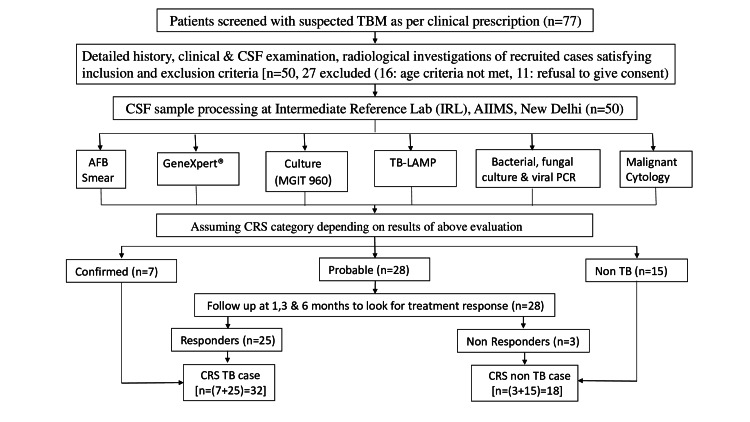
Workflow of the study CSF: cerebrospinal fluid; AFB: acid-fast bacilli; TB-LAMP: loop-mediated isothermal amplification; PCR: polymerase chain reaction; CRS: composite reference slandered

A prospective observational study was conducted at All India Institute of Medical Sciences in New Delhi, India, from January 2019 to December 2020 after obtaining ethical clearance from the institute's ethical committee. Fifty subjects suspected to be having TBM based on inclusion criteria were included after obtaining written informed consent. Inclusion criteria were age more than 14 years with fever for five or more days with one or more of the following: vomiting (projectile), neck stiffness, convulsions, altered consciousness, focal neurological deficit, or headache. In addition, all subjects underwent clinical examination, cerebrospinal fluid (CSF) examination (including microbiological tests), and brain imaging. Based on the above evaluation, patients were assigned a composite reference standard (CRS) category: confirmed, probable, or non-TB. They were further divided into CRS TB and CRS non-TB cases for final analysis after follow-up, as depicted in Figure 1.

CRS is an established rule used to make a final diagnosis depending on the outcome of two or more tests, called component tests. In this study, clinical features, CSF findings, AFB staining, GeneXpert®, MGIT 960 culture, brain imaging, and response to anti-tubercular treatment (ATT) constituted the CRS. All patients were assigned a CRS category which was defined as follows: The first is confirmed: culture or AFB positive with clinical features suggestive of TBM and started on ATT were included as CRS TB cases. The second is probable: culture and AFB negative but clinical features suggestive of TBM along with GeneXpert positive or CSF biochemical examination and brain imaging representative of TBM, followed up at one, three, and six months to look for ATT response. Based on the response, these were further divided into CRS TB and CRS non-TB cases. The third is non-TB: negative culture, AFB, and all other tests for TB, and the patient did not receive ATT. These were included as CRS non-TB cases.

TB-LAMP was performed using 60 microliters of CSF, as per the manufacturer's instructions [12]. We used a multitarget molecular assay targeting two MTB (*Mycobacterium tuberculosis*)-specific sequences in the DNA, insertion sequence 6110 (IS6110) and gyrase subunit B (*gyrB*), manufactured by Eiken Chemical Company Limited (Tokyo, Japan). All CSF samples were processed at the Intermediate Reference Laboratory (IRL), an accredited mycobacteriology lab for the diagnosis of TB, certified by the Central TB Division, Government of India.

Ethical clearance

The study was approved by the Institute Ethics Committee for Postgraduate Research of All India Institute of Medical Sciences (AIIMS), New Delhi (approval number: IECPG-30/23.01.2019 dated 25.01.2019).

Statistical analysis

All patients' characteristics, CSF results, imaging findings, and treatment responses after six months of ATT were integrated for the final clinical diagnosis as per CRS. The diagnostic efficacy of TB-LAMP against CRS, MGIT 960 culture, and GeneXpert® was assessed by calculating sensitivity, specificity, and positive and negative predictive values (PPV and NPV) using standard formulas. In addition, Cohen's kappa value was calculated to look for agreement between TB-LAMP, MGIT 960 culture, and GeneXpert®. The kappa value varies between -1 and +1, and the higher the kappa value, the better the agreement between the two tests. A p-value of <0.05 was considered statistically significant.

## Results

**Figure 2:**
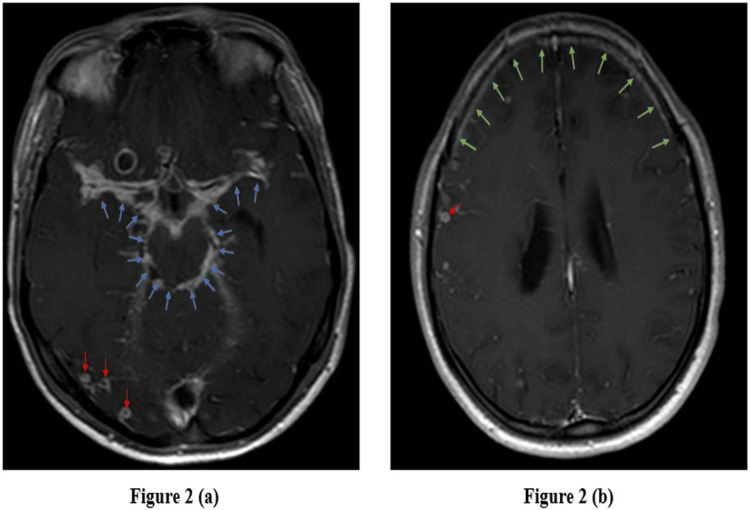
Axial post-gadolinium contrast T1-weighted MR images of the brain Axial post-gadolinium contrast T1-weighted MR images of the brain showing basal meningeal enhancement (marked by blue arrows) in (a) and diffuse meningeal enhancement (marked by green arrows) in (b) in a TBM patient. Also noted are multiple ring and nodular enhancing lesions (marked by red arrows) suggestive of tuberculomas. TBM: tuberculous meningitis

**Figure 3:**
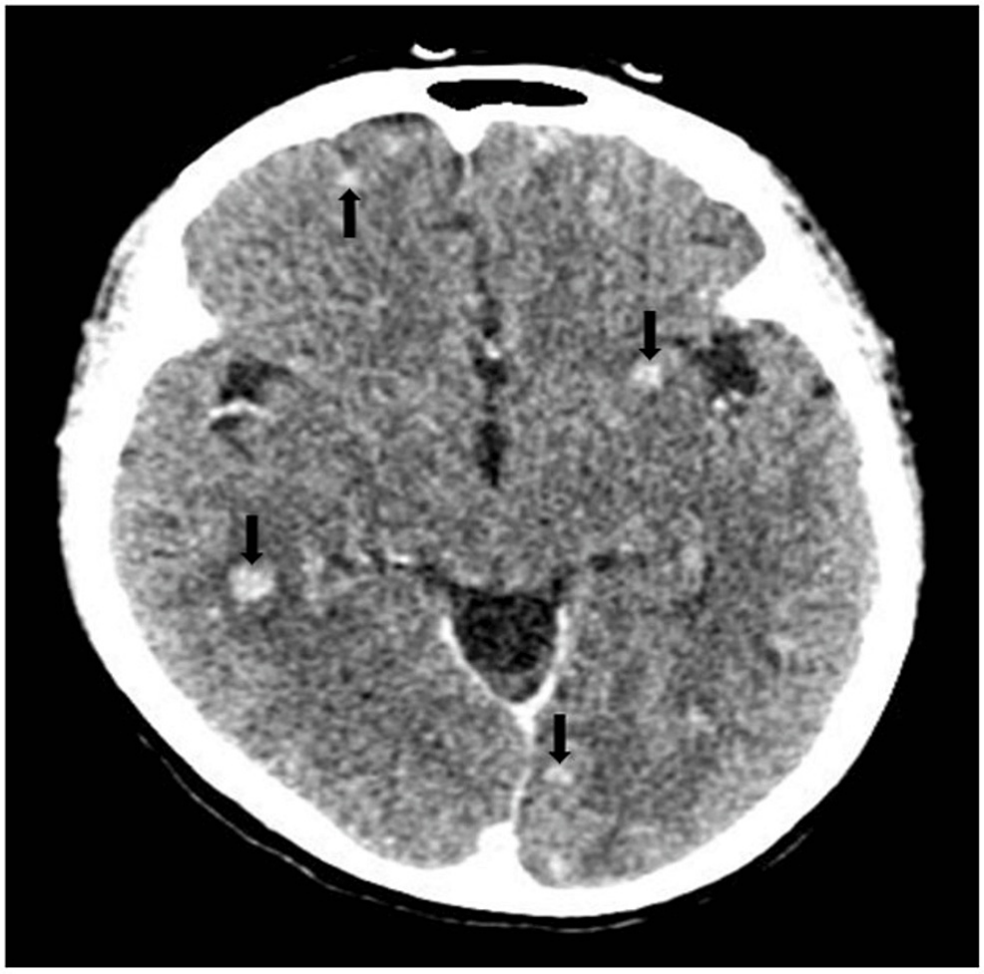
Axial CECT scan Axial CECT scan of the brain showing multiple tuberculomas (marked by black arrows) with variable surrounding white matter edema in a TBM patient. CECT: contrast-enhanced computed tomography; TBM: tuberculous meningitis

**Figure 4:**
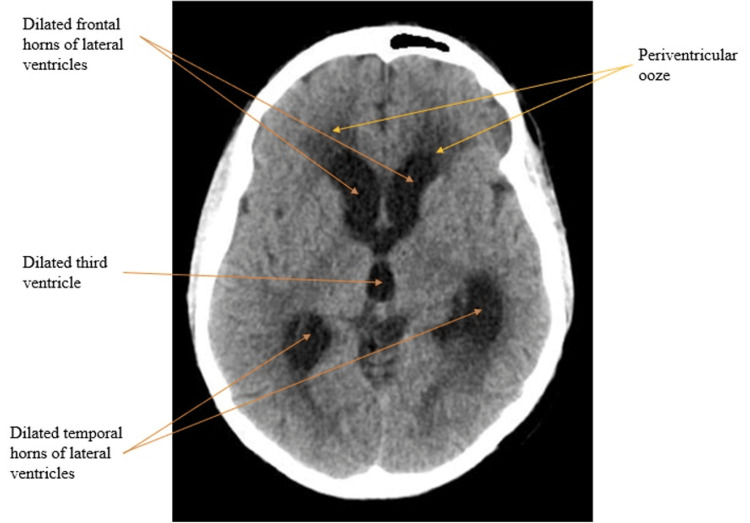
Axial NCCT scan Axial NCCT scan of the brain showing dilated frontal and temporal horns of the lateral ventricle and dilated third ventricle along with periventricular ooze suggestive of hydrocephalus in a TBM patient. NCCT: non-contrast computed tomography; TBM: tuberculous meningitis

Fifty patients were there in the final analysis whose clinical characteristics have been displayed in Table 1. The mean age of the study participants was 31.5±15.8 years, with 66% of them being males. The most common comorbidity was hypertension, present in 12% of the patients. Among symptoms and signs, headache (86%) and altered sensorium (74%) were the most common, followed by neck rigidity (46%). CSF pleocytosis was seen in 60% of the patients, while lymphocyte predominance was seen in 46%. CSF protein was increased to more than 100 mg/dl in 58% of the patients, while 36% had hypoglycorrhachia. Brain imaging was abnormal in 88% of the patients, with basal meningeal enhancement (38%) being the most frequent finding, followed by tuberculomas (36%) and hydrocephalus (24%), respectively. Representative images of these common brain imaging findings have been depicted in Figure 2, Figure 3, and Figure 4, respectively. Out of 50 patients, 32 were CRS TB cases, and 18 were CRS non-TB cases, as per criteria. Among 18 CRS non-TB cases, eight had infective etiology with one case each of bacterial and cryptococcal meningitis and three cases each of viral meningitis and septic encephalopathy. Eight CRS non-TB cases were of inflammatory etiology with one case each of neuro-Behcet's, acute disseminated encephalomyelitis (ADEM), autoimmune encephalitis, neuromyelitis optica spectrum disorder (NMOSD), CNS focal angiitis, and idiopathic hypertrophic meningitis and two cases of CNS lupus. Two CRS non-TB cases were of malignant etiology, one was a case of CNS leukemia, and the other was a case of brain metastasis secondary to lung cancer. The sensitivity of CSF, AFB staining, TB-LAMP, and GeneXpert® for TBM diagnosis against CRS was 3% (1/30), 53% (17/32), and 53% (17/32), respectively. The specificity of AFB staining, TB-LAMP, and GeneXpert® against CRS was 100% (16/16), 78% (14/18), and 89% (16/18), respectively.

**Table 1:** Clinical characteristics CSF: cerebrospinal fluid; HIV: human immunodeficiency virus

Demographic variable			Total (n=50) N (%)
Mean age (years)			31.5±15.8
Male (%)			33 (66.0)
Comorbidities		Hypertension	6 (12.0)
		Diabetes mellitus	2 (4.0)
		HIV	1 (2.0)
		Others	9 (18.0)
Symptoms and signs	Headache		43 (86.0)
	Altered sensorium		37 (74.0)
	Vomiting		24 (48.0)
	Focal neurologic deficit		10 (20.0)
	Convulsions		8 (16.0)
	Neck rigidity		23 (46.0)
CSF parameters	Pleocytosis (>5 cells/microliter)		32 (64.0)
	Pleocytosis (>10 cells/microliter)		30 (60.0)
	Lymphocyte predominance		23 (46.0)
	Elevated protein (>45 mg/dl)		40 (80.0)
	Elevated protein (>100 mg/dl)		29 (58.0)
	Hypoglycorrhachia (low sugar)		18 (36.0)
Brain imaging	Abnormal		44 (88.0)
	Basal meningeal enhancement		19 (38.0)
	Tuberculoma		18 (36.0)
	Hydrocephalus		12 (24.0)
	Infarct		7 (14.0)
	Other findings		14 (28.0)

On comparing TB-LAMP against CRS, the diagnostic performance of LAMP was as follows: sensitivity 53% (17/32), specificity 78% (14/18), PPV 81% (17/21), and NPV 48% (14/29), as shown in Table 2, and there was a fair agreement between the two, which was statistically significant (Cohen's kappa=0.27 and p=0.034).

**Table 2:** Comparison of TB-LAMP against CRS (n=50) CSF: cerebrospinal fluid; TB: tuberculosis; LAMP: loop-mediated isothermal amplification; CRS: composite reference standard; PPV: positive predictive value; NPV: negative predictive value; CI: confidence interval

		CRS			Sensitivity (95% CI)	Specificity (95% CI)	PPV (95% CI)	NPV (95% CI)
		Positive	Negative	Total				
CSF: TB-LAMP	Positive	17 (34.0%)	4 (8.0%)	21 (42.0%)	53% (35-71)	78% (52-94)	81% (63-91)	48% (37-59)
	Negative	15 (30.0%)	14 (28.0%)	29 (58.0%)				
	Total	32 (64.0%)	18 (36.0%)	50 (100.0%)				

On comparing GeneXpert® against CRS, the diagnostic performance of GeneXpert® was as follows: sensitivity 53% (17/32), specificity 89% (16/18), PPV 90% (17/19), and NPV 52% (16/31), as shown in Table 3, and there was a fair agreement between the two methods, which was statistically significant (Cohen's kappa=0.36 and p=0.003).

**Table 3:** Comparison of GeneXpert against CRS (n=50) CSF: cerebrospinal fluid; CRS: composite reference standard; PPV: positive predictive value; NPV: negative predictive value; CI: confidence interval

		CRS			Sensitivity (95% CI)	Specificity (95% CI)	PPV (95% CI)	NPV (95% CI)
		Positive	Negative	Total				
CSF: GeneXpert	Positive	17 (34.0%)	2 (4.0%)	19 (38.0%)	53% (35-71)	89% (65-99)	90% (69-97)	52% (42-61)
	Negative	15 (30.0%)	16 (32.0%)	31 (62.0%)				
	Total	32 (64.0%)	18 (36.0%)	50 (100.0%)				

On comparing TB-LAMP against culture, the diagnostic performance of LAMP was as follows: sensitivity 100% (6/6), specificity 64% (16/25), PPV 40% (6/15), and NPV 100% (16/16), as shown in Table 4, and there was a moderate agreement between the two methods, which was statistically significant (Cohen's kappa=0.41 and p=0.005).

**Table 4:** Comparison of TB-LAMP against culture (n=31) CSF: cerebrospinal fluid; TB: tuberculosis; LAMP: loop-mediated isothermal amplification; PPV: positive predictive value; NPV: negative predictive value; CI: confidence interval; MGIT: mycobacteria growth indicator tube

CSF		Culture (MGIT)			Sensitivity (95% CI)	Specificity (95% CI)	PPV (95% CI)	NPV
		Positive	Negative	Total				
TB-LAMP	Positive	6 (19.4%)	9 (29.0%)	15 (48.4%)	100% (54-100)	64% (43-82)	40% (28-53)	100%
	Negative	0 (0.0%)	16 (51.6%)	16 (51.6%)				
	Total	6 (19.4%)	25 (80.6%)	31 (100.0%)				

On comparing TB-LAMP against GeneXpert®, the diagnostic performance of LAMP was as follows: sensitivity 95% (18/19), specificity 90% (28/31), PPV 86% (18/21), and NPV 97% (28/29), as shown in Table 5, and there was almost perfect agreement between the two methods, which was statistically significant (Cohen's kappa=0.83 and p<0.001).

**Table 5:** Comparison of TB-LAMP against GeneXpert (n=50) CSF: cerebrospinal fluid; TB: tuberculosis; LAMP: loop-mediated isothermal amplification; PPV: positive predictive value; NPV: negative predictive value; CI: confidence interval

CSF		GeneXpert			Sensitivity (95% CI)	Specificity (95% CI)	PPV (95% CI)	NPV (95% CI)
		Positive	Negative	Total				
TB-LAMP	Positive	18 (36.0%)	3 (6.0%)	21 (42.0%)	95% (74-99.9)	90% (74-98)	86% (67-95)	97% (81-99.5)
	Negative	1 (2.0%)	28 (56.0%)	29 (58.0%)				
	Total	19 (38.0%)	31 (62.0%)	50 (100.0%)				

## Discussion

Timely diagnosis of TBM is challenging because of the paucibacillary nature of the disease, which accounts for the low sensitivity of conventional microscopy and slow turnover time of culture, often leading to delay in diagnosis and giving rise to high rates of death and disability. Thus, there is a need for a test that has good diagnostic efficacy with minimal requirements that can be used in resource-limited peripheral settings. TB-LAMP assay is a novel DNA amplification method that is temperature independent and provides results within an hour, in the form of a visual display that can be read with the naked eye under ultraviolet light and needs minimal infrastructural requirements, thus making it ideal for use in peripheral (rural) areas of low-income countries like India for TBM diagnosis [13-14].

Various studies have demonstrated TB-LAMP's role in pulmonary TB diagnosis. The WHO has recommended it for the diagnosis of pulmonary TB in adults with symptoms suggestive of disease. TB-LAMP can also be used as a follow-up test to AFB smear in adults with clinical suspicion of pulmonary Koch's, especially when further testing of sputum-negative samples is necessary [15].

Data on the role of TB-LAMP assay in the diagnosis of TBM is scarce. Furthermore, the use of TB-LAMP assay in TBM diagnosis is not well standardized; therefore, the present study was planned to evaluate the role of multitargeted TB-LAMP assay in TBM diagnosis.

In the study, the diagnostic performance of AFB staining against CRS was as follows: sensitivity, 3% (1/30) and specificity, 100% (16/16). The sensitivity in our study was lower than the reported sensitivity of about 10-20% in patients with extrapulmonary TB, especially TBM [16]. This was mainly due to the low mycobacterial load in CSF of TBM patients. The sensitivity can be increased by obtaining a large CSF volume (10 ml), several samples examined over a few days, centrifuging it at around 3000 rpm and carefully looking at slides by an adequately trained person for 30 minutes [17]. However, the applicability of these methods is impractical in clinical settings. However, the results were comparable to a few studies that also have reported low sensitivity of CSF AFB staining like 2.91% reported by Sun et al. and 4% reported by Modi et al. [18,19].

Comparison of GeneXpert® against CRS showed a sensitivity of 53% and specificity of 89%. A meta-analysis and systematic review were done by Pai et al., which reported GeneXpert sensitivity and specificity to be 56% and 98%, respectively, which is comparable to our study [20]. On comparing TB-LAMP against culture, the sensitivity was 100%, along with a specificity of 64%. The sensitivity of TB-LAMP was comparable to the sensitivity reported by Modi et al. against culture (96%). However, specificity was lower (64% versus 100%) as a culture could be done only for 31 of the study participants due to the scarcity of volume of CSF available for testing [19]. The sensitivity and specificity of TB-LAMP against CRS were found to be 53% and 78%, respectively, which were similar to GeneXpert® against CRS (53% and 89%), with specificity being slightly lower for TB-LAMP. The results were comparable to a study done by Sun et al., with sensitivity being somewhat better in our study (53% versus 43%) and specificity being slightly lower (78% versus 92%) as instead of an in-house LAMP assay; they also used the LoopAMP MTBC assay, a commercial assay [18,21]. The better sensitivity of our study can be explained by the fact that the assay we used targeted two genes, namely, *gyrB* and IS6110, compared to the single target gene (IS6110) used by them [18]. However, Nagdev et al. [22] reported a higher sensitivity of TB-LAMP (88%) in TBM diagnosis with an almost similar specificity of 80%. Similarly, Modi et al. reported a higher sensitivity of 88% and specificity of 100% for TB-LAMP in TBM diagnosis [19]. Possible reasons for the difference in results can be the use of in-house LAMP assays in both the studies by Modi et al. and Nagdev et al., the use of different target genes IS6110 and MPB64 by Modi et al. and only IS6110 by Nagdev et al., and difference in the sample size of studies (50 in our study, 27 in the study by Nagdev et al. [21], 250 in the study by Modi et al. [19]) [19,22]. A comparison of the diagnostic efficacy of various microbiological tests of our study with existing literature has been summarized in Table 6.

**Table 6:** Comparison of the diagnostic efficacy of various microbiological tests in our study with existing literature AFB: acid-fast bacilli; PCR: polymerase chain reaction; MGIT: mycobacteria growth indicator tube; TB-LAMP: LoopAMP™ MTBC kit

S. no.	Study	CSF (microbiological test)	Diagnostic efficacy
1	Our study	AFB smear	Sensitivity: 3%
		MGIT	Sensitivity: 22%
		GeneXpert	Sensitivity: 53% and specificity: 89%
		TB-LAMP	Sensitivity: 53% and specificity 78%
2	[22]	Nested PCR	Sensitivity: 52.9% and specificity: 90%
		TB-LAMP	Sensitivity: 88.23% and specificity: 80%
3	[19]	AFB smear	Sensitivity: 4%
		PCR	Sensitivity: 74.6% and specificity: 100%
		TB-LAMP	Sensitivity: 88% and specificity: 100%
4	[18]	AFB smear	Sensitivity: 2.91%
		MGIT	Sensitivity: 12.79%
		PCR	Sensitivity: 34.3%
		TB-LAMP	Sensitivity: 43.02% and specificity: 92.86%
5	[16]	AFB smear	Sensitivity: 10-20%
6	[17,23]	MGIT	Sensitivity: 60-70%

Comparing TB-LAMP against GeneXpert® for TBM diagnosis, there was almost perfect agreement between the two methods with a kappa value of 0.83, a high sensitivity of 95%, and a high specificity of 90%. Thus, TB-LAMP may be used in place of GeneXpert®, where the implementation of GeneXpert® is restricted by laboratory infrastructure and continuous power supply requirements. The WHO Guideline Development Group had also suggested that in peripheral centers, TB-LAMP could be implemented as a microscopy replacement test where implementation of GeneXpert® is restricted by requirements of continuous power supply and infrastructure [15]. In cost and affordability analysis, done in Vietnam and Malawi for the nationwide implementation of GeneXpert® and TB-LAMP as a routine service in peripheral centers, the weighted average per test cost was around 20.06-26.86 USD for GeneXpert and 14.37-15.85 USD for LAMP [23,24]. Therefore, the weighted average per test cost for LAMP was almost two-thirds of GeneXpert, thus making it cost-effective and suitable for low-income countries like India. However, the disadvantage of using TB-LAMP in place of GeneXpert is a lack of information regarding rifampicin susceptibility.

It was a prospective study having both TBM (confirmed and probable) and non-TBM cases, which gave a better idea about the diagnostic efficacy of TB-LAMP. The follow-up was done prospectively to include response to ATT, and probable cases were further categorized into TBM cases and non-TBM cases based on clinical response. The diagnostic efficacy of TB-LAMP was evaluated against GeneXpert® on the same samples. The sample size was a sample of convenience (50) because logistic reasons may be one of the limitations of this study. In addition, the positivity rate of CSF culture was low (around 20% (6/31)) owing to the limited volume of CSF samples being available for testing, due to which culture could only be done for 62% (31/50) patients.

## Conclusions

The performance of the TB-LAMP assay is comparable to GeneXpert® in diagnosing TBM and may play a role at point-of-care level in resource-limited and peripheral settings. Irrespective of other NAATs, TB-LAMP does not provide any information regarding drug-resistant TB. However, LAMP can be used as a replacement for conventional microscopy for both pulmonary and extrapulmonary specimens specially in the case of TBM. Therefore, in low-income high-disease-burden countries, it can be used as an alternative to GeneXpert® owing to its good diagnostic efficacy, minimal requirements, and ability to produce rapid results. TB-LAMP should be further explored in various types of specimens to be established as key diagnostics in various settings.
